# Marine heatwaves shape size‐dependent thermal exposure, habitat use and marine residency in Arctic char (*Salvelinus alpinus*)

**DOI:** 10.1111/jfb.70543

**Published:** 2026-06-23

**Authors:** Jessica E. Desforges, David Côté, Joey Angnatok, J. Brian Dempson, Travis E. Van Leeuwen

**Affiliations:** ^1^ Northwest Atlantic Fisheries Centre, Fisheries and Oceans Canada St. John's Newfoundland and Labrador Canada; ^2^ Putjotik Fisheries Ltd. Nunatsiavut Newfoundland and Labrador Canada

**Keywords:** acoustic telemetry, habitat use, marine heatwave, marine residency, migration timing, size‐dependent response

## Abstract

Marine heatwaves (MHWs) are increasing in high‐latitude oceans, yet behavioural responses of anadromous fishes to these potential stressors during short marine feeding seasons remain poorly understood. We combined acoustic telemetry (internal temperature and depth) with satellite‐derived sea surface temperature data to quantify Arctic char (*Salvelinus alpinus*) responses to MHWs in the coastal Labrador Sea across two contrasting summers: 2023, with frequent early‐season MHWs, and 2024, dominated by a late‐season event. Using non‐MHW detections, we fit seasonal baselines for each fish and calculated temperature and depth anomalies. Char tended to occur in inner habitats (estuaries/fjords), though 2024 showed greater among‐individual variability in outer (coastal/headland) habitat use. Internal temperatures increased relative to baseline during MHW onset (median +0.64°C) with modest persistence post‐onset (+0.23°C), whereas depth anomalies showed no consistent departures. Temperature anomalies increased from pre to during MHWs across body sizes and were strongest for larger fish during onset. In contrast, MHW conditions increased the probability of inner‐habitat detection primarily for smaller fish after controlling for seasonality. Migration timing differed between years in a size‐dependent way, with larger fish leaving the marine environment and entering rivers earlier in 2023. Overall, Arctic char responses did not provide evidence that they sought out thermal refuges during MHWs. However, size‐dependent shifts in habitat use and migration timing indicate that MHWs can still alter marine habitat trade‐offs, likely through indirect ecological factors (e.g., prey availability). Arctic char responses may intensify, however, when future heatwaves exceed physiological or growth‐performance thresholds.

## 1 INTRODUCTION

The Arctic is warming at four times the global average (Rantanen et al., 2022), and record‐breaking temperature anomalies have been reported across Arctic and Subarctic marine environments (Frölicher & Laufkötter, 2018; Guinaldo & Neukermans, 2026). Marine heatwaves (MHWs) are discrete periods of anomalously warm water (Pearce et al., 2011), typically identified when sea surface temperatures (SST) exceed a seasonally varying threshold (90th percentile) for at least five consecutive days (Hobday et al., 2016). These events have become more frequent and intense in recent years, particularly in high‐latitude marine ecosystems (Oliver et al., 2018; Oliver et al., 2021).

The nearshore coastal Labrador Sea—an important marine feeding area for anadromous Arctic char (*Salvelinus alpinus*)—experienced severe heatwaves in summer 2023, with temperatures exceeding historical averages for extended periods (Guinaldo & Neukermans, 2026; Moustafa et al., 2023). Warming occurred concurrently across marine and freshwater environments (Geissinger et al., 2024), and rivers exceeded lethal limits for salmonids (>24°C) on multiple occasions, prompting widespread angling closures and raising concerns about thermal stress in migratory species (Geissinger et al., 2024).

Arctic char display substantial variability in life history strategies (Klemetsen et al., 2003; Reist et al., 2013), morphology (O'Connell & Dempson, 2002) and diet (Côté et al., 2021; Dempson et al., 2002), and have historically persisted across a wide range of environmental conditions. Despite this flexibility, anadromous populations near the southern extent of their range are expected to be increasingly vulnerable under climate change projections (Layton et al., 2021; Reist et al., 2006). Although direct studies linking MHWs to the behaviour and distribution of wild Arctic char remain limited, warmer temperatures associated with MHWs are emerging as major stressors (Ballinger et al., 2024; Geissinger et al., 2024; Guinaldo & Neukermans, 2026) and, through exposure to high temperatures, are predicted to influence survival, migration strategy and timing (Gilbert et al., 2016), habitat selection (Côté et al., 2021; Jensen et al., 2014; Nordli et al., 2023), feeding ecology (Anderson et al., 2023; Falardeau et al., 2022), growth (Gunnarsson et al., 2011) and spawning dynamics (Rikardsen et al., 2007).

After overwintering in freshwater, adult Arctic char migrate to the marine environment and spend the summer feeding in prey‐rich coastal waters. This marine phase is critical for accumulating energy reserves and typically lasts 30–45 days, although much shorter feeding periods have been documented (e.g., 6 days; Dutil, 1986; Gyselman, 1994; Harris et al., 2020). Recent studies and local Inuit knowledge have linked warming conditions to longer ice‐free seasons and the appearance of new marine species, which may improve fish condition through increased lipid accumulation over time (Falardeau et al., 2022). Warming‐induced dietary shifts have been attributed to increased pelagic prey availability when Arctic char enter the ocean, potentially driven by earlier spring blooms, northward expansion of boreal species and warmer coastal waters that may facilitate greater use of deeper pelagic areas (Anderson et al., 2023; Falardeau et al., 2022). In some systems, recent climatic conditions may remain below physiological thresholds for Arctic char (15–20°C; Gilbert et al., 2020; Gilbert & Tierney, 2018) and could instead enhance thermal performance by improving feeding conditions and growth opportunities. Several studies report growth optima to occur within 11–14°C, with some degree of population‐level variation (Beuvard et al., 2022; Larsson et al., 2005). For example, internal body temperatures of Arctic char in the Kitikmeot Sea have reached 13.2°C while SSTs were ~8°C (Falardeau et al., 2022), approaching temperatures associated with increased post‐exercise fatigue, elevated metabolic demand and reduced growth efficiency (Gilbert et al., 2020; Gilbert & Tierney, 2018; Larsson & Berglund, 2005). Similar internal‐external temperature offsets have also been documented in other Arctic char telemetry studies (Mulder et al., 2020). Internal temperatures exceeding SST, but remaining within optimal thermal ranges, may therefore reflect occupancy of warmer microhabitats that provide foraging advantages or support growth optimization.

Across their marine range, Arctic char can access diverse thermal habitats, from productive nearshore estuaries to cooler, deeper coastal and pelagic waters. Many studies report predominant use of nearshore environments such as estuaries (Côté et al., 2021; MacMillan‐Kenny et al., 2025; Moore et al., 2016; Spares et al., 2015), likely due to rich foraging opportunities (Miller & Sadro, 2003), proximity to river systems (Dempson & Kristofferson, 1987), highly productive diatom‐rich habitats (MacMillan‐Kenny et al., 2025) and refuge from predators (Jensen & Rikardsen, 2012), although utilization can vary substantially among years (Côté et al., 2021). In contrast, stable isotope evidence indicates that exploitation of pelagic resources may be associated with higher body condition in years with reduced sea ice and anomalously high SSTs (Anderson et al., 2023; Falardeau et al., 2022), suggesting that warm conditions could reduce reliance on nearshore habitats if pelagic prey becomes more available or profitable. However, responses may also occur in the opposite direction: Dubos et al. (2023) documented increased nearshore habitat use during warmer conditions in Ungava Bay, where local knowledge emphasized the benefits of complex nearshore habitats for thermal refuge and predator avoidance, particularly for smaller individuals (Dubos et al., 2023). Together, these findings indicate that warming can alter nearshore versus outer‐habitat use, but the direction of the shift may depend on local habitat structure, prey abundance and individual traits. Importantly, warmer conditions may not always represent thermal stress for Arctic char; under current conditions, moderate warming may move some marine habitats closer to temperatures associated with optimal growth or performance before physiological thresholds are exceeded.

Thermal preference, habitat use and diet are also expected to be size‐dependent, with larger individuals potentially adopting different strategies than smaller conspecifics during periods of abnormal warming (Elliott & Elliott, 2010; Magnuson et al., 1979; Morita et al., 2010; Mulder et al., 2020). In salmonids, optimal temperatures for growth generally decline with body size, such that smaller individuals often prefer warmer habitats than larger individuals (Jonsson & Jonsson, 2011; Morita et al., 2010). In Labrador, Mulder et al. (2020) found that larger Arctic char used significantly cooler waters than smaller individuals, suggesting ontogenetic shifts in thermal habitat use consistent with growth optimization. Consequently, high SSTs associated with increasingly prevalent MHWs may not only drive dietary or habitat shifts but also elicit size‐dependent movement strategies that maintain body temperatures within an optimal range for performance.

Collectively, this literature suggests a narrow, size‐dependent thermal margin (Deutsch et al., 2008; Huey et al., 2012; Sunday et al., 2014) between conditions that enhance performance and those that may exceed physiological limits and reduce growth efficiency. It also highlights the potential for Arctic char to modify movement and habitat use in response to short‐term thermal variability, yet direct evidence linking MHWs to behavioural responses during the marine feeding period remains scarce. Here, we combine acoustic telemetry with satellite‐derived SSTs to address this knowledge gap. First, we examined the relationship between internal body temperature and SST to determine whether elevated SSTs represent relevant metrics for MHW classifications. This analysis also provided context for whether fish‐experienced temperatures approached published growth‐performance reference values (~14°C). We then tested whether Arctic char experienced warmer conditions during MHWs by estimating seasonal baselines of internal temperature from non‐MHW detection data. We hypothesized that internal temperatures would increase during MHWs if fish continued to occupy warm near‐surface waters and exploit thermal conditions favourable for feeding and growth. However, we expected the opposite to be true should MHW SSTs exceed optimal thermal conditions, where char may seek thermal refuge in cooler deep or coastal waters. Second, we examined whether depth use deviated from seasonal expectations during MHWs. We predicted that char would generally maintain depth use near baseline to sustain foraging efficiency, unless temperatures approached physiological limits that would favour behavioural thermoregulation through changes in depth use. Third, we tested whether behavioural anomalies differed among event phases defined relative to heatwave onset, and whether any changes evident during onset persisted into the post‐MHW phase. Fourth, we quantified size‐dependent shifts in inner (estuaries and fjords) versus outer (coastal headlands) habitat use during MHW phases while controlling for seasonal movement patterns. We predicted that larger fish, with greater swimming capacity and different thermal optima, would be more likely to exploit outer habitats to access pelagic prey or maintain cooler temperatures conducive to growth during MHWs. If fish‐experienced temperatures during MHWs remain within growth‐performance ranges, we hypothesized that there would be no evidence supporting size‐dependent preference for outer habitats, instead persisting in warmer waters to capitalize on growth. Finally, we compared migration timing between 2023—a summer characterized by frequent, early onset MHWs—and 2024, when MHWs occurred later in the season, to evaluate how cumulative heat exposure may influence the duration of the marine feeding period. We expected shorter marine residencies under warmer, efficient growth temperatures, with larger individuals meeting energetic requirements earlier than smaller conspecifics. In comparison, we hypothesized that 2024 might experience a relatively delayed upstream migration, as fish continue to gather energetic reserves, particularly during the MHWs later in the summer.

## 2 METHODS

### 2.1 Study area

The waters surrounding the community of Nain, Nunatsiavut, Labrador, have supported healthy populations of Arctic char for several centuries (Brice‐Bennett et al., 1977; J. Angnatok, personal communication) and a robust commercial fishery for decades (Coady, 1974; Dempson, 1995; Dempson et al., 2008). This approximately 50 km wide area is characterized by several rivers and deep, freshwater fjords, where char are often found during the winter (Côté et al., 2021). These freshwater systems lead to shallow estuaries and deeper saltwater habitat along rocky islets nearing open ocean, where char feed during the summer months. Bathymetry data suggest saltwater habitats reach beyond 110 m in coastal environments (MacMillan‐Kenny et al., 2025; Nutt, 1953), while depths of over 200 m have been recorded in freshwater habitat, specifically Tasisuak Lake, a well‐known Arctic char overwintering site among locals (J. Angnatok, personal communication).

**FIGURE 1:**
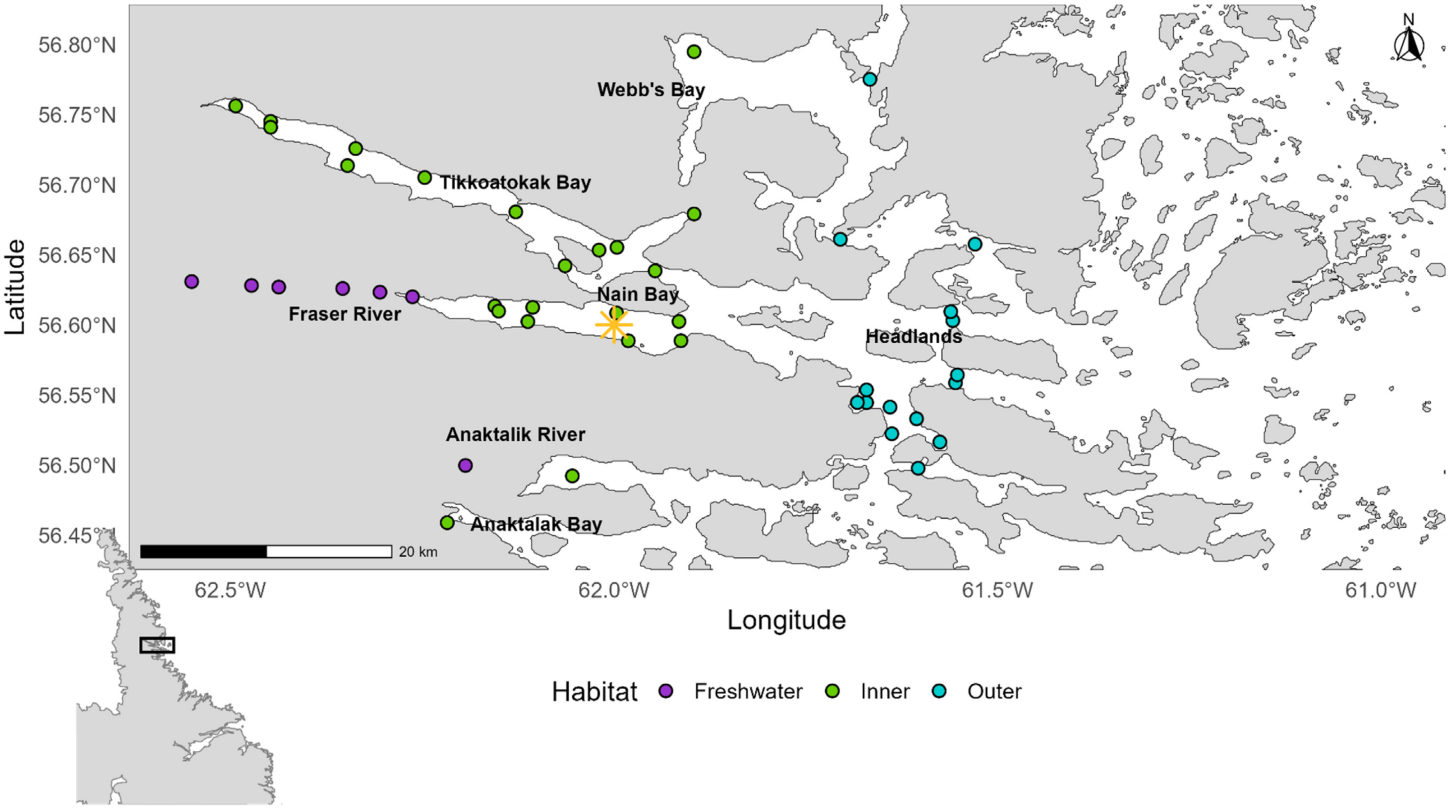
Receiver deployment locations across the study area in northern Labrador, Canada. Acoustic receivers were classified into 3 habitat categories based on their spatial characteristics and salinity profile: Freshwater (purple), Inner habitat (green) and Outer habitat (blue). The main map shows the detailed placement of receivers used to detect Arctic char (*Salvelinus alpinus*) movements during their summer marine migration. An inset map (lower left) highlights the location of the study area relative to the broader Labrador coastline, showing proximity to the Labrador Sea and the open ocean. The star represents the reference location for sea surface temperatures (SSTs) extracted from Copernicus.

Habitat categories were defined by classifying the study area into three primary types, freshwater, inner (includes estuaries, fjords) and outer (includes coastal islets, headlands). Estuaries were defined as shallow habitats near freshwater outflows, while fjords comprised deeper, sheltered inlets leading into estuarine areas. Coastal habitats were the most saline and included regions near offshore islets leading to the open ocean. From 2022 to 2024, 46 omnidirectional 69‐kHz acoustic receivers (VEMCO VR2W and VR2AR) were deployed across the area to monitor habitat use and marine migration timing (Figure 1). Seven receivers were located in freshwater habitats (receiver depth range 5–75 m, mean 16 m), 23 in inner marine habitats (5–107 m, mean 47 m) and 16 in outer marine habitats (8–94 m, mean 40 m). Receivers were moored approximately 1–2 m above the bottom. Receiver locations were selected to monitor movements between freshwater, estuarine/fjord and coastal habitats during the marine feeding period.

### 2.2 Fish tagging and detection filtering

A total of 49 Arctic char were captured in the Fraser River and surgically implanted with V13TP acoustic transmitters equipped with temperature and depth sensors (tag life approximately 585 days, nominal delay 110–190 s, maximum depth 34 m). Fish were captured by angling with single barbless‐hook lures or by gillnet with 11.5‐cm mesh. Fish were anaesthetized in a clove oil solution (1 part clove oil to 9 parts ethanol; 40 mg/L), measured for fork length (mm), weighed (g) and placed on a sterilized V‐shaped surgical table supplied with flow‐through river water. A 1.5–2 cm incision was made anterior to the pelvic girdle, the tag was inserted into the abdominal cavity and the incision was closed with one or two independent sutures. Fish recovered in a flow‐through holding pen and were monitored for approximately 1 h before release near the capture location. The care and use of experimental animals complied with Canadian Council on Animal Care animal welfare laws, guidelines and policies as approved by the Northwest Atlantic Fisheries Centre Animal Care Committee under the Animal Use Protocol NAFC‐2022‐06. Fish sizes and sample sizes by tagging period are summarized in Table 1.

**TABLE 1:** Summary of Arctic char tagging numbers, dates and release sites.

Release date	Release site	Latitude	Longitude	No. of fish tagged	Fork length (mean ± SD, mm)	Mass (mean ± SD, g)	No. of fish retained	No. of detections
1–2 Oct 2022	Fraser River	56.63116° N	−62.5045° W	12	416 ± 36.8	746 ± 223.5	8	117,094
15 May 2023	Fraser River	56.61972° N	−62.2536° W	20	455 ± 37.9	790 ± 170.6	17	207,342
22–23 Aug 2023	Fraser River	56.62511° N	−62.4068° W	17	444 ± 38.5	1057 ± 459.9	12	319,319

*Note*: Numbers of tagged fish retained for further analyses and total number of detections for each given tagging period are also shown.

Receiver detections were processed using the residency() function in the R package actel (version 1.3.0, Flávio & Baktoft, 2021). False detections were filtered by requiring at least two valid detections overall and at least two detections per valid movement event. Movement and depth plots were then inspected to identify biologically implausible records, extended inactivity between arrays, or abnormal vertical patterns. Eleven fish were removed after detection screening, and one additional fish was removed because internal temperatures near 36°C with an abnormal depth profile suggested depredation (Figure S1). The final behavioural dataset included 36 fish.

### 2.3 Marine heatwave detection

**FIGURE 2:**
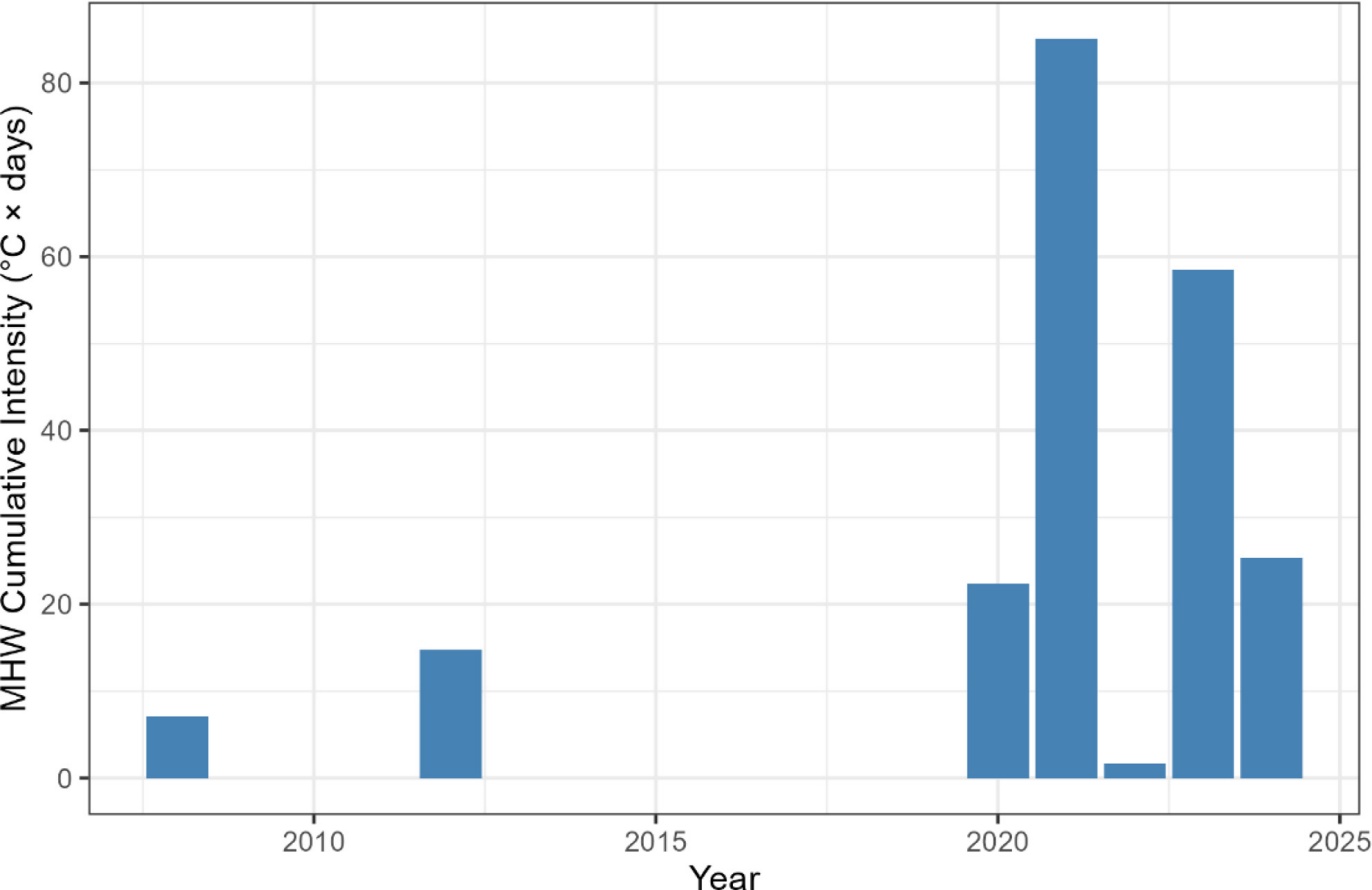
Cumulative marine heatwave (MHW) intensity during the Arctic char (*Salvelinus alpinus*) marine feeding season (May–August) for each year from 2007 to 2024. Cumulative intensity (°C × days) was calculated as the sum of daily sea surface temperature (SST) anomalies above the 90th percentile threshold for all marine heatwave days observed for a given year from May to August to reflect the marine residency period.

Daily SST data were obtained from the Copernicus Marine Environment Monitoring Service product METOFFICE‐GLO‐SST‐L4‐NRT‐OBS‐SST‐V2 (Copernicus Marine Service, 2022). SST was extracted from a fixed regional reference location near the center of the study area (56.589° N, 61.929° W) from 1 January 2007 to 1 November 2024 (Figure 2). This location was selected to represent regional surface thermal conditions within the marine migration corridor, while recognizing that local temperatures within estuaries, fjords and coastal habitats may differ from this surface reference.

MHWs were identified using heatwaveR (Schlegel & Smit, 2018). A daily climatology and 90th‐percentile seasonal threshold were generated with ts2clm() using 2007–2022 as the climatology period. Events were detected with detect_event() and defined as periods of at least five consecutive days when SST exceeded the seasonally varying 90th‐percentile threshold. We summarized the number and proportion of MHW days during the primary marine‐season window (1 June–30 August) in 2023 and 2024 and also calculated cumulative MHW days through the season. Because early‐season heat exposure was expected to be especially relevant to the marine feeding period, we also summarized MHW exposure during an early window (1 June–15 July).

### 2.4 Data preparation and daily summaries

Behavioural analyses were based on daily summaries. For each fish and calendar date, receiver‐level median internal body temperature, median depth and detection counts were first summarized. When fish were detected at multiple receivers on the same day, internal temperature and depth were combined into a single daily value using detection‐weighted means of receiver‐level medians. This produced one record per fish per date for anomaly analyses, avoided duplicate fish‐days and aligned the behavioural data with daily SST‐derived MHW classifications. We considered summarizing fish‐recorded internal temperatures by habitat and depth stratum; however, detections were too sparse and unevenly distributed across habitat‐depth combinations to support robust habitat‐depth inference. As a descriptive context only, we summarized fish‐recorded internal temperatures by inner versus outer marine habitat and month. These summaries represent temperatures experienced by fish in used habitats, not fixed environmental water‐temperature measurements.

Habitat use was summarized from the same daily detection records. For each fish‐day with marine detections, we retained the number of detections classified to inner and outer marine habitats (*n*_inner and *n*_outer). Freshwater detections were excluded from inner‐versus‐outer habitat‐use models because those models were intended to describe marine habitat choice. We refer to these summaries as detection‐weighted habitat use or proportions of classified marine detections, rather than time spent, because detection counts can be affected by receiver coverage and detection conditions. This one‐record‐per‐day summarization is commonly used in acoustic telemetry analyses when individuals can be recorded across multiple receivers within short time intervals (Gutowsky et al., 2017; Mulder et al., 2020).

To assess whether the regional SST time series used to identify marine heatwaves reflected the thermal conditions experienced by tagged Arctic char, we conducted a descriptive SST–fish temperature comparison restricted to marine detections during June–August 2023 and 2024 using SST values from the fixed regional SST extraction point. We quantified the SST‐fish temperature relationship using Pearson correlations at two scales: the fish‐day scale and the calendar‐day scale, where fish temperatures were averaged across all individuals detected in marine habitat on a given date. We also fit a descriptive mixed‐effects model with fish internal temperature as the response, SST as the predictor and fish identity as a random intercept. This analysis was used to evaluate whether regional SST captured broad variation in fish thermal experience during the marine feeding period, but it was not intended as a calibration between satellite SST and habitat‐specific water temperature because implanted tags measure internal body temperature and fish detections represent selected locations.

### 2.5 Seasonal baselines and behavioural anomalies

To evaluate behavioural departures from expected seasonal patterns, we estimated seasonal baselines for internal temperature and depth and calculated anomalies as observed minus predicted values (Hobday et al., 2016). Baselines were fit preferentially using non‐MHW fish‐days so that expected seasonal behaviour was not driven by heatwave periods. Temperature anomalies were calculated as *T*_anom = *T* − *T*_pred, and depth anomalies as *D*_anom = *D* − *D*_pred, where *T* and *D* are observed daily internal temperature and depth, and *T*_pred and *D*_pred are predicted seasonal baselines. Positive *T*_anom values indicate warmer internal temperatures than expected for a given fish and date, and positive *D*_anom values indicate deeper use than expected.

We used a two‐tier baseline approach. For fish with adequate seasonal coverage, per‐fish harmonic regressions (Bhaskaran et al., 2013; Cornelissen, 2014) were fit to describe expected seasonal variation in temperature or depth as a function of day of year, with year included when an individual was observed in more than 1 year. For fish with sparse coverage or unstable per‐fish predictions, we used pooled hierarchical generalized additive models with a cyclic day‐of‐year smoother and random effects for fish and year. Detailed eligibility thresholds, smoothness checks and model equations are provided in the Supplementary Methods. The selected baseline method was stored for each individual and used to generate predicted temperature and depth for all fish‐days.

### 2.6 Heatwave event alignment and phase assignment

To examine behavioural change around MHW onset, fish‐days were aligned to the first day of each detected MHW event. For each event, we defined a 43‐day window extending from 21 days before to 21 days after event onset and assigned relative day t_rel as the number of days from event start. Fish‐days were classified into three phases: Pre (t_rel < 0), During (0 ≤ t_rel ≤ 7) and Post (t_rel > 7). When windows around nearby MHW events overlapped, each date was assigned to the closest event onset to ensure that each fish‐date occurred only once in the phase‐labelled dataset; exact ties were assigned to the earlier event. Fish‐days outside all event windows were excluded from phase‐based analyses.

### 2.7 Statistical analyses

We used complementary analyses to evaluate thermal exposure, depth use, habitat use and migration timing. First, paired within‐fish contrasts were used as a robust summary of anomaly responses to MHW phases. For each fish, mean *T*_anom and *D*_anom were calculated within each phase and paired differences were calculated for During minus Pre and Post minus Pre. Across‐fish effects were summarized as median paired differences, with 95% confidence intervals (CIs) estimated by nonparametric bootstrap resampling of fish.

Second, daily temperature and depth anomalies were modelled using linear mixed‐effects models with fixed effects of phase, standardized fork length and their interaction. Fish identity was included as a random intercept to account for repeated measures, and year was included as a random intercept when more than 1 year was represented. Estimated marginal means and phase contrasts were evaluated at representative body sizes (length_sc = −1, 0, and +1 SD).

Third, inner‐versus‐outer marine habitat use was modelled with a binomial generalized additive mixed model using cbind(*n*_inner, *n*_outer) as the response. Fixed effects included MHW status, standardized fork length and their interaction. A cyclic smooth of day of year accounted for seasonal movement structure, and random‐effect smooths accounted for repeated observations of fish and interannual variation. Model predictions were reported as the probability of inner‐habitat detection, P(inner), for small, mean‐sized and large fish, with MHW versus non‐MHW contrasts presented as odds ratios.

Finally, migration timing was indexed using non‐freshwater detections to reduce sensitivity to differences in freshwater receiver coverage. For each fish‐year, marine entry was defined as the first non‐freshwater detection and return/upstream timing as the last non‐freshwater detection within the seasonal window. Fish‐years were retained when the first non‐freshwater detection occurred on or before 30 June and the span between first and last non‐freshwater detections exceeded 20 days. Last non‐freshwater day of the year was modelled as a function of year, standardized fork length and their interaction. A fish‐level random intercept was evaluated but was omitted from the final inference when the fitted variance was effectively zero. All statistical analyses were conducted in R (R Core Team, 2026) using tidyverse (Wickham et al., 2019) for data handling and visualization, lubridate (Grolemund & Wickham, 2011) for date‐time manipulation, mgcv (Wood, 2011) for baseline models and lme4 (Bates et al., 2015) and emmeans (Lenth, 2025) for mixed‐effects modelling and post hoc estimation.

## 3 RESULTS

### 3.1 SST, behaviour and distribution

During June–August 2023–2024, the marine fish‐day dataset contained 749 valid fish‐day records from 26 individuals, distributed across 163 calendar dates with at least one marine detection. Fish‐recorded internal temperature was positively associated with regional SST. At the fish‐day scale, internal temperature and SST were moderately correlated (Pearson's *r* = 0.50, *p* ≤ 0.0001, Figure S2). When fish temperatures were averaged across individuals within each calendar date, the positive association remained and was slightly stronger (*r* = 0.60, *p* ≤ 0.0001; Figure S2). A descriptive mixed‐effects model with fish identity as a random intercept similarly indicated that fish internal temperature increased with regional SST, with an estimated increase of 0.38°C in fish internal temperature for each 1°C increase in SST (95% CI: 0.33–0.43°C; *p* ≤ 0.0001). These results indicate that the regional SST time series used to identify MHWs captured broad variation in Arctic char thermal experience during the marine feeding period, although the relationship was not one‐to‐one.

**FIGURE 3:**
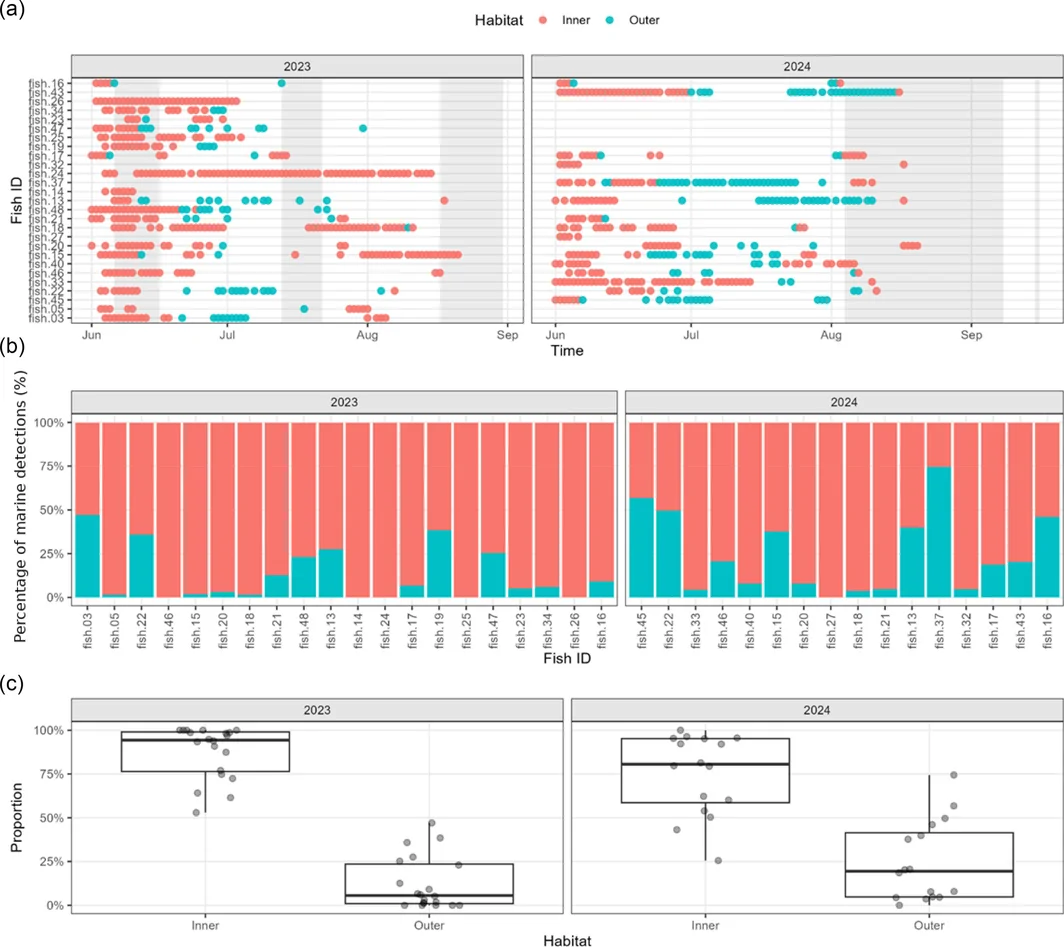
Seasonal inner–outer habitat use of anadromous Arctic char (*Salvelinus alpinus*) during the marine period in 2023 and 2024, with marine heatwave (MHW) periods highlighted. (a) Fish‐day habitat timelines showing the dominant marine habitat (inner vs. outer) used by each tagged individual across the marine season. Grey vertical bands indicate days classified as MHW conditions based on the sea surface temperature (SST) heatwave detection. (b) Detection‐weighted percentage of marine detections in inner versus outer habitat for each individual within each year. (c) Among‐fish distribution of individual‐level proportions of detections in inner and outer habitat within each year (boxplots show median and interquartile range; points are individual fish). Only marine detections classified to inner or outer habitat were included; freshwater detections were excluded.

Across both years, tagged Arctic char displayed strong individual variation in marine habitat use but were generally inner‐biased during marine residency. Fish‐day timelines (Figure 3a) showed prolonged periods of inner habitat occupancy for many individuals, punctuated by intermittent bouts of outer habitat use. Detection‐weighted summaries indicated that most fish had the majority of their marine detections assigned to inner habitats in 2023, whereas 2024 exhibited greater among‐individual variability, including several fish with substantial outer‐habitat detections (Figure 3b). Consistent with these patterns, the distribution of individual‐level habitat proportions was more concentrated toward high inner use in 2023 and more dispersed in 2024 (Figure 3c). 2023 also featured more frequent MHWs during the typical at‐sea marine feeding period compared to 2024, which was characterized by one prolonged MHW near the end of the marine phase. MHW periods occurred within the marine season in both years (grey bands, Figure 3a) and overlapped with periods of inner and outer occupancy, providing a seasonal context for subsequent event‐window analyses of phase‐specific habitat responses.

Heatwave exposure differed primarily in timing between years. During the early window (1 June–15 July), 2023 experienced substantial MHW exposure (13 MHW days; 28.9% of days) arising from two discrete events, whereas 2024 exhibited no MHW days and no events during the same period. Across the full marine‐season window (1 June–30 August), the total proportion of MHW days was broadly comparable between years (2023: 33/91 days, 36.3%; 2024: 27/91 days, 29.7%), but the seasonal accumulation differed: MHW days accrued early in 2023 (first event start 6 June) and were concentrated late in 2024 (first event start 4 August), consistent with a year characterized by frequent early‐season heatwaves (2023) versus a year dominated by late‐season heatwave exposure (2024, Figure 4).

**FIGURE 4:**
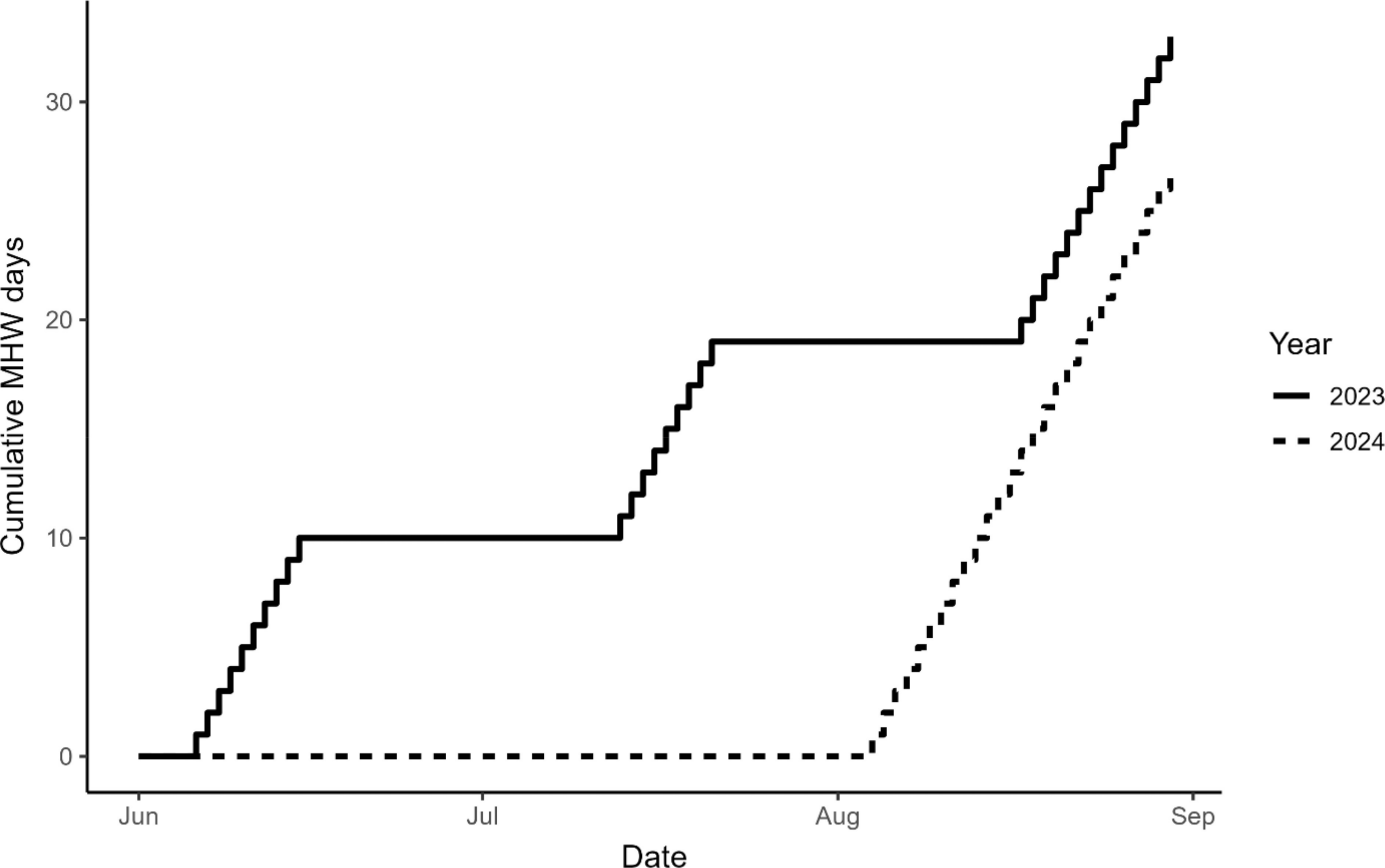
Cumulative number of marine heatwave (MHW) days during the marine‐season window (1 June–30 August) in 2023 and 2024. Curves increase only on days classified as MHW (heatwaveR definition), illustrating differences in the timing of heatwave exposure across years (early‐season accumulation in 2023 vs. late‐season accumulation in 2024).

### 3.2 Seasonal baselines and behavioural anomalies

**FIGURE 5:**
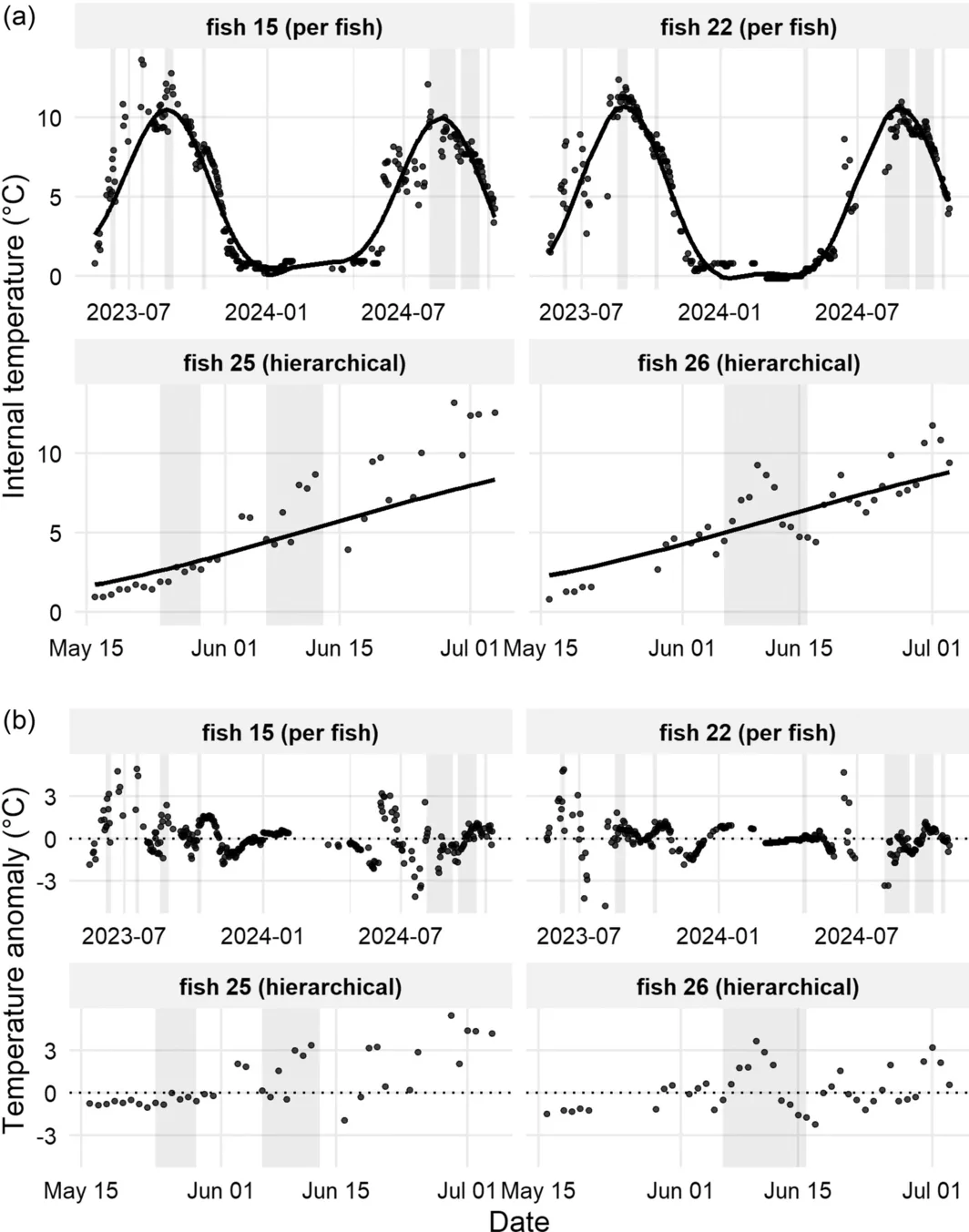
Seasonal baseline fitting and anomaly calculation for four example Arctic char (*Salvelinus alpinus*) (fish 15 (per fish); fish 22 (per fish); fish 25 (hierarchical); fish 26 (hierarchical)). (a) Observed daily internal body temperature (points) and predicted seasonal baseline (line). Grey shading indicates days classified as marine heatwave (MHW) conditions. (b) Temperature anomalies relative to the baseline (*T*_anom = *T* − *T*_pred); the dashed line indicates zero anomaly. *X*‐axes are free within panels to show each individual's available detection window.

Behavioural analyses were conducted on daily summaries (one record per individual per calendar day). After collapsing detections to fish‐day means, the dataset used for baseline fitting comprised 3787 fish‐days across 36 individuals (2022–2024), including 663 fish‐days classified as MHW conditions. Seasonal baselines for internal temperature and depth were fit preferentially at the individual level using per‐fish harmonic regression when fish had sufficient non‐MHW coverage and day‐of‐year span. Twenty‐four fish met these criteria and were used to generate per‐fish baselines, while 12 fish with sparse coverage were used within hierarchical pooled baselines. Baseline predictions were smooth over time; for example, the median maximum day‐to‐day change in predicted temperature was 0.40°C for per‐fish baselines and 0.15°C for hierarchical baselines, indicating that expected seasonal curves did not exhibit abrupt day‐to‐day swings (see Figure 5 for four examples).

The resulting phase‐labelled anomalies dataset included 2168 fish‐days (Pre: 813; During: 626; Post: 729), representing 36 fish in Pre, 33 fish in During and 32 fish in Post. Paired within‐fish phase summaries (fish‐level means within phase) showed that internal temperatures were consistently warmer relative to baseline during heatwave windows. The median within‐fish change in temperature anomaly from pre to during was +0.64°C (bootstrapped 95% CI: 0.46–0.79, *n* = 34 fish). The median within‐fish change from pre to post was smaller but remained positive (+0.23°C, 95% CI: 0.09–0.39, *n* = 32 fish), suggesting modest persistence beyond the first week of the event window. In contrast, paired within‐fish depth use contrasts provided no evidence of consistent depth departures from baseline during or after heatwaves (Pre to During: +0.06 m, 95% CI: −0.27 to 0.31, *n* = 33 fish; Pre to Post: −0.03 m, 95% CI: −0.26 to 0.19, *n* = 32 fish).

**FIGURE 6:**
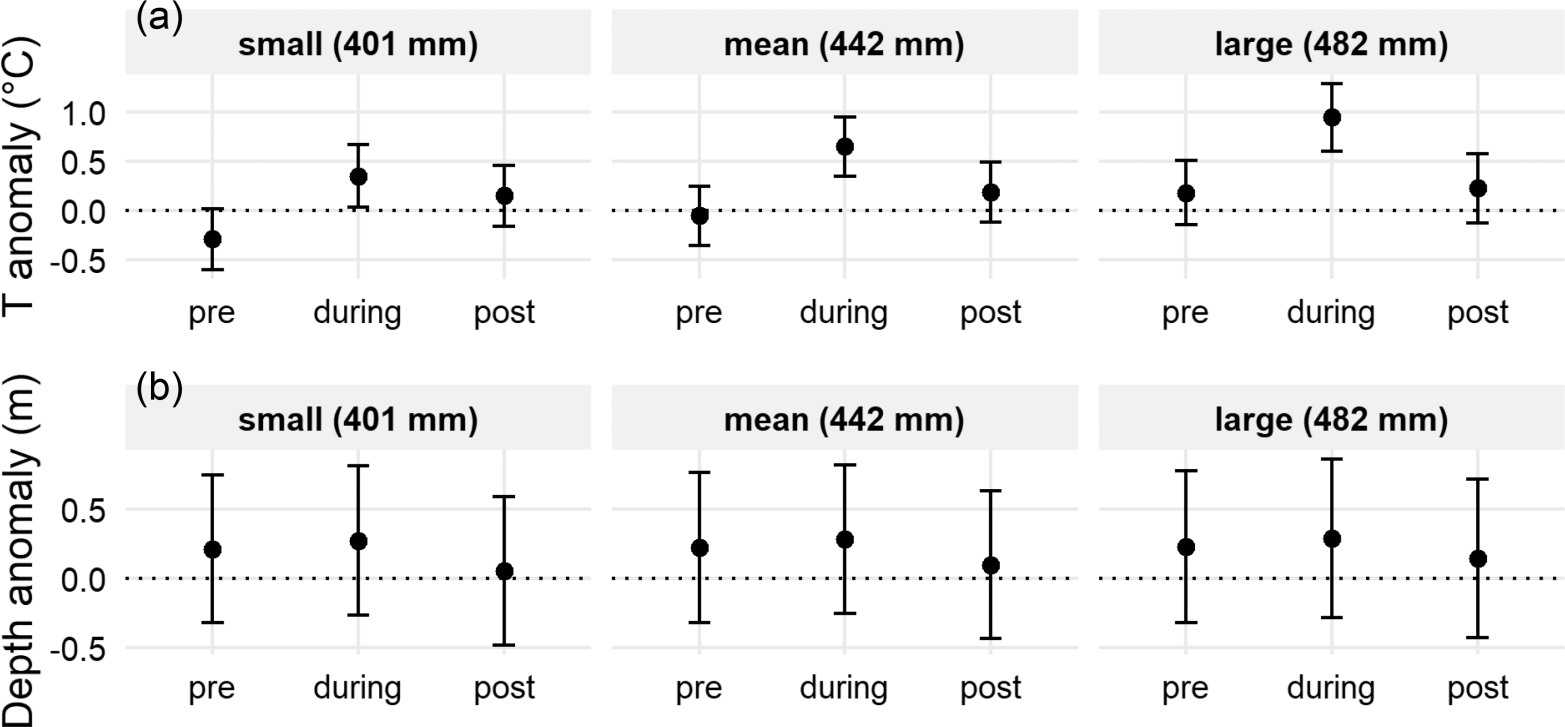
Model‐predicted behavioural anomalies in Arctic char (*Salvelinus alpinus*) across heatwave phases as a function of body size. Points show model‐predicted marginal means (predicted anomalies) [±95% confidence interval (CI)] from linear mixed‐effects models fit to daily anomalies with fixed effects phase × standardized length and random intercepts for fish (and year when applicable). Length is evaluated at three reference values (−1 SD, mean, +1 SD; approximately 401, 442 and 482 mm). (a) Temperature anomalies (*T*_anom). (b) Depth anomalies (*D*_anom).

Daily anomaly mixed‐effects models showed clear differences among phases and a phase‐by‐length effect for temperature anomalies (2106 fish‐days from 36 fish; random effect for year included). Model‐estimated marginal means indicated that temperature anomalies increased from Pre to During at all representative body sizes (−1 SD = 401 mm, mean = 442 mm, +1 SD = 482 mm). For small fish (−1 SD), predicted temperature anomalies were −0.29°C (Pre), +0.35°C (During) and +0.15°C (Post). For mean‐sized fish, predicted anomalies were −0.06°C (Pre), +0.56°C (During) and +0.39°C (Post). For large fish (+1 SD), predicted anomalies were +0.18°C (Pre), +0.94°C (During) and +0.22°C (Post). Tukey‐adjusted contrasts indicated that during exceeded Pre at all sizes (all *p* ≤ 0.0001). Post exceeded pre at small and mean size (*p* = 0.004 and *p* = 0.0003, respectively), but Pre and Post did not differ at large size (*p* = 0.92). Length effects also varied by phase: anomalies increased with length in Pre (*p* = 0.016) and During (*p* = 0.0003), but showed no detectable length dependence in Post (*p* = 0.65). Together, these results indicate that warming relative to baseline peaked during the first week after event onset and was most pronounced for larger individuals, whereas post‐anomalies were weaker and less size‐structured (Figure 6).

### 3.3 Habitat use

**FIGURE 7:**
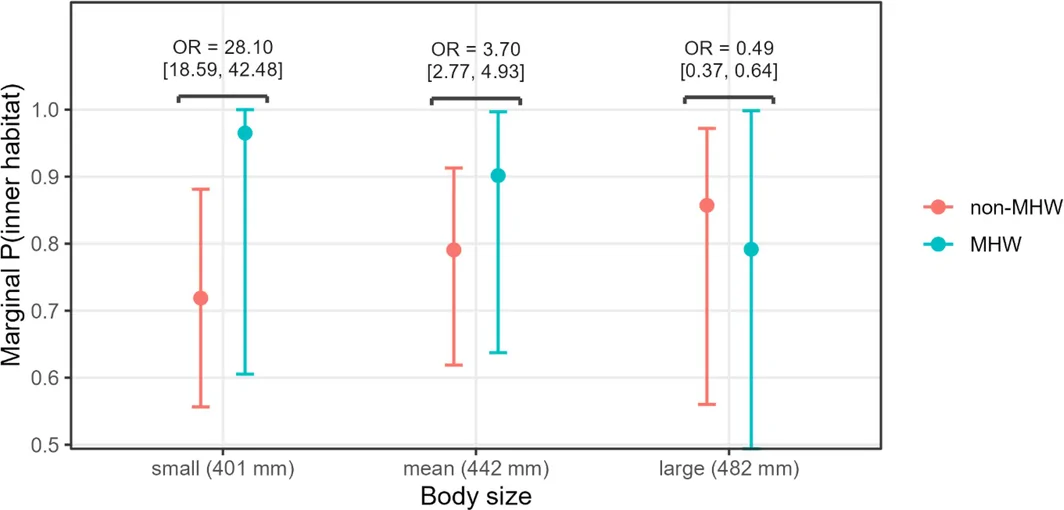
Season‐controlled, marginal standardized effects of marine heatwave (MHW) days on inner‐habitat use across body size. Points show the marginal mean probability of inner‐habitat use, P(inner), predicted from a binomial generalized additive mixed model fit to daily detection counts (cbind(*n*_inner, *n*_outer); logit link) with fixed effects of MHW status × standardized fork length (length_sc), a cyclic smooth of day‐of‐year (DOY) to account for seasonal structure (s(DOY), bs = ‘cc’), and random‐effect smooths for fish ID (and year). Marginal means were obtained by averaging predicted probabilities over the observed DOY distribution and excluding fish/year random effects (population‐level predictions). Error bars show 95% cluster‐bootstrap confidence intervals for the marginal means (resampling fish). Body size is shown at −1 SD, mean and +1 SD fork length (approximately 401, 442 and 482 mm). Brackets and labels report the formal MHW versus non‐MHW contrast evaluated at each size on the logit scale as an odds ratio (OR) with 95% confidence limits. OR >1 indicates higher odds of inner‐habitat use on MHW days; OR <1 indicates lower odds.

Habitat use differed between MHW and non‐MHW days in a size‐dependent way (*N* = 798 fish‐days), even after accounting for strong seasonal structure in habitat use (smooth of DOY: edf = 7.94, *p* ≤ 0.0001). The fixed effect of MHW status was positive (*β* = 1.308 ± 0.147, *p* ≤ 0.0001) while the MHW × length interaction was negative (*β* = −2.028 ± 0.103, *p* ≤ 0.0001), indicating that the MHW‐associated increase in inner‐habitat detection was strongest for smaller fish and weakened with increasing body size. Consistent with this interaction, marginal predictions showed higher P(inner) on MHW days for small and mean‐sized fish, whereas the effect reversed for large fish (Figure 7). Although 95% intervals on the plotted marginal means can overlap, especially where MHW days are sparse and uncertainty is large, formal model contrasts on the logit scale supported clear differences at the evaluated sizes [odds ratio for MHW vs. non‐MHW: small = 28.1 (18.6, 42.5), mean = 3.70 (2.77, 4.93), large = 0.49 (0.37, 0.64)], showing a MHW‐associated increase in inner‐habitat detections for smaller fish but reduced inner‐habitat odds for the largest individuals once seasonality was controlled.

### 3.4 Migratory behaviour

**FIGURE 8:**
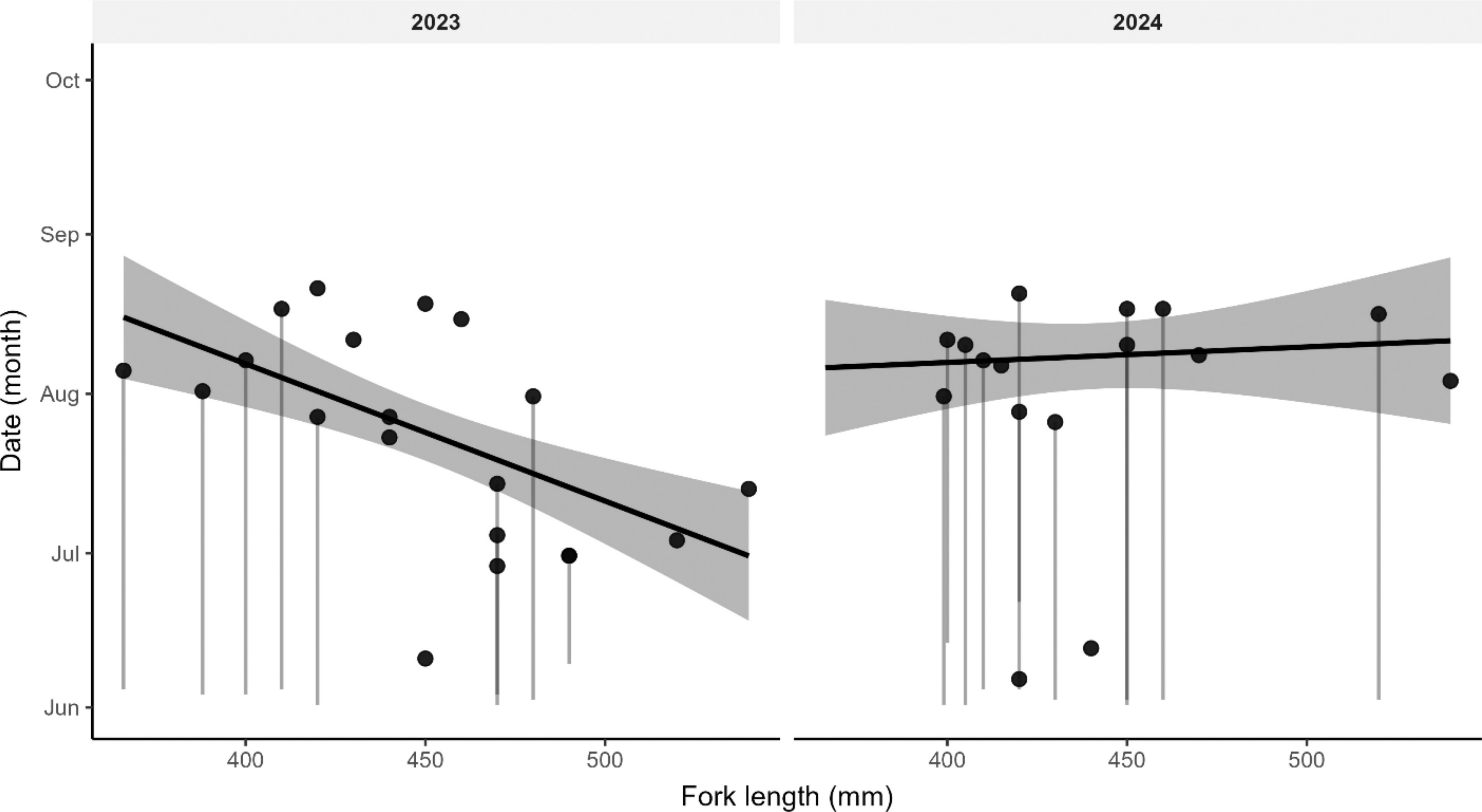
Size‐dependent variation in the timing of last non‐freshwater detections (proxy for upstream migration) for tagged Arctic char (*Salvelinus alpinus*) in 2023 and 2024. Points denote the last non‐freshwater date for each fish‐year (aligned to a reference year for display). Vertical lines indicate the observed span of non‐freshwater detections (first to last non‐freshwater date) for fish‐years that met minimum residency criteria (first non‐freshwater date ≤June 30; residency duration >20 days; and ≥minimum non‐freshwater detection days). Fish‐years plotted without vertical lines did not meet these criteria (or lacked an observed first non‐freshwater date) and are shown as points only. Solid lines show fitted values from the year × length linear model of last non‐freshwater day‐of‐year; shaded bands denote 95% confidence intervals.

After applying migratory‐event filters (first non‐freshwater detection on or before June 30 and a non‐freshwater ‘feeding span’ >20 days), the dataset comprised 33 fish‐years from 25 individuals (2023: 19 fish‐years; 2024: 14 fish‐years). Median first non‐freshwater detections occurred around early June (2023: May 31; 2024: June 2), whereas median last non‐freshwater detections occurred earlier in 2023 (July 27) than in 2024 (Aug 9), with corresponding median feeding spans of 60 and 66.5 days, respectively. A subset of 7 fish‐years had last non‐freshwater detections before July 15 (all in 2023) but still met the feeding‐span criterion (22–46 days). In modelling last non‐freshwater timing (DOY), the year × length interaction was supported over the additive model (AIC 264 vs. 271), indicating size‐dependent interannual differences. In the interaction model, 2024 fish were detected later than 2023 fish at mean size (year effect = +14.16 days, SE = 4.32, *p* = 0.0027), and size effects differed between years: in 2023, larger fish tended to have earlier last non‐freshwater detections (length effect = −11.66 days per +1 SD length, SE = 2.82, *p* = 0.000275), while this relationship was substantially reduced in 2024 (year × length = +12.98 days, SE = 4.41, *p* = 0.0063). Interaction‐aware estimated marginal means showed negligible year differences for small fish (−1 SD; contrast = −1.18 days, *p* = 0.85), but significantly later last non‐freshwater timing in 2024 for mean‐sized fish (0 SD; +14.16 days, *p* = 0.0027) and especially large fish (+1 SD; +27.13 days, *p* = 0.0002), indicating that earlier termination of non‐freshwater detections in 2023 was driven primarily by larger individuals (Figure 8).

## 4 DISCUSSION

MHWs are increasing in frequency and intensity in high‐latitude systems (Oliver et al., 2021), and Arctic coastal environments are warming rapidly (Rantanen et al., 2022). Our results show that MHW conditions can alter the thermal experience and behaviour of anadromous Arctic char during their short marine feeding season. Across years, fish were generally inner‐biased during the marine season, while interannual differences in MHW timing coincided with differences in habitat use and marine departure. Internal body temperatures increased relative to seasonal baselines during the first week after MHW onset, whereas depth anomalies showed little consistent departure. This pattern suggests that fish often experienced warmer conditions during the onset rather than fully buffering exposure via depth use. The lack of consistent depth anomalies suggests that Arctic char did not avoid MHW‐associated warming through vertical movement. Instead, fish appeared to tolerate the elevated thermal exposure, consistent with the interpretation that current MHW intensities remained within a range that Arctic char could accommodate during the marine feeding period. Whether this represents an adaptive increase in thermal opportunity (e.g., improved foraging efficiency and growth potential) or a constraint‐driven tolerance of pervasive warming likely depends on local habitat structure and prey dynamics, which can vary across systems and years. Because Arctic char enter the marine environment primarily to feed, movement and habitat shifts during MHWs may be driven not only by direct thermal exposure, but also indirectly through changes in prey availability, distribution and phenology. Heatwaves can alter the timing and location of productive foraging opportunities (e.g., nearshore vs. more pelagic prey fields, see Anderson et al., 2023), potentially changing the profitability of inner and outer habitats independent of behavioural thermoregulation per se. Under this view, thermal anomalies may partly reflect where fish must forage during heatwave conditions rather than a simple preference for warmer or cooler water. Disentangling these pathways will require concurrent prey indicators or foraging proxies. Habitat responses to MHW conditions were strongly size dependent: smaller fish increased inner‐habitat use on MHW days after controlling for seasonality, whereas this MHW‐associated shift weakened with body size and was absent or reversed for the largest individuals. Interannual differences in the timing of marine departure were also size dependent, with earlier termination of marine detections by large fish in 2023 than in 2024. Together, these findings indicate that acute warming events can reshape thermal exposure and habitat trade‐offs in a size‐dependent way, with potential consequences for marine residency and energetic outcomes.

Across both years, tagged Arctic char were generally inner‐biased, showing prolonged occupancy of inner habitats punctuated by intermittent bouts of outer habitat use. This pattern is consistent with telemetry studies documenting predominant nearshore and estuarine use by anadromous Arctic char during the marine phase (Côté et al., 2021; Harris et al., 2020; Moore et al., 2016; Spares et al., 2015). Inner habitats may be energetically profitable during a short feeding window because they provide high foraging opportunities (Miller & Sadro, 2003), proximity to freshwater migration routes (Dempson & Kristofferson, 1987), favourable salinity and temperature conditions (Harris et al., 2020) and refuge from predators (Jensen & Rikardsen, 2012). At the same time, we observed among‐individual variability, particularly in 2024, when several fish exhibited substantial outer habitat use. Such variability is consistent with evidence that habitat use can shift among years (Côté et al., 2021) and may reflect interannual differences in prey fields, hydrography, or the relative profitability of pelagic resources.

Importantly, heatwave exposure differed mainly in timing between years: in 2023, MHWs occurred early within the typical marine feeding period, whereas in 2024, heatwave exposure accumulated primarily later in the season. This distinction matters because early‐season warming can coincide with rapid seasonal transitions in prey availability and primary productivity (Falardeau et al., 2022), when fish are accumulating energy reserves, while late‐season heatwaves may occur closer to freshwater return. Biological impacts of MHWs are often strongly contingent on event timing relative to key seasonal stages (Hobday et al., 2016; Oliver et al., 2021), and this seasonal context likely contributes to the contrasting patterns observed here.

Internal temperatures were consistently warmer than expected during heatwave onset windows, indicating that fish experienced elevated thermal conditions beyond what would be expected from seasonal progression alone. This finding aligns with the general expectation that acute warming events can shift the thermal landscape available to mobile ectotherms (Hobday et al., 2016) and complements prior work showing that Arctic char internal temperatures can exceed SST, potentially reflecting use of warmer habitats to support feeding or growth optimization (Falardeau et al., 2022).

Temperature anomalies also showed a clear size structure during heatwave onset. Larger fish exhibited stronger warming relative to baseline during the event window, whereas post‐onset anomalies were smaller and less consistently size‐dependent. Several mechanisms could produce this pattern. Larger fish may exploit different foraging habitats or activity regimes during onset, either by intensifying use of warm inner habitats during peak feeding opportunities or by moving through warmer water masses while foraging. Ontogenetic differences in thermal preference and performance may also contribute (Mulder et al., 2020). In salmonids, optimal growth temperatures generally decline with body size (Jonsson & Jonsson, 2011; Morita et al., 2010), and Arctic char in Labrador show size‐dependent thermal habitat use, with larger fish tending to occupy cooler waters overall (Mulder et al., 2020). If larger individuals have lower thermal optima or narrower performance margins, strong warming during onset coupled with weaker persistence post‐onset may reflect rapid behavioural compensation (e.g., shifting habitat use or activity) to return toward preferred thermal conditions after the initial heatwave period.

These patterns reinforce an important conceptual point: temperature anomalies indicate exposure, not necessarily stress. Depending on how close experienced temperatures are to growth‐optimizing ranges, elevated internal temperatures could enhance digestion and growth potential or increase metabolic costs and reduce efficiency (Gilbert et al., 2020; Gilbert & Tierney, 2018; Larsson & Berglund, 2005). Fish‐recorded internal temperatures in both inner and outer habitats generally remained below the 14°C growth‐performance reference value reported for Arctic char (Figure S3), suggesting that temperatures experienced during the observed heatwaves remained within or near ranges associated with growth opportunity rather than acute thermal limitation. Distinguishing short‐term exploitation of favourable conditions from avoidance of costly conditions will require linking thermal exposure to prey availability, activity and growth proxies (e.g., bioenergetics modelling or condition metrics).

In contrast to internal temperature, depth anomalies showed no consistent departures from seasonal expectations during or after heatwaves. This suggests that behavioural responses were not expressed as systematic, population‐wide depth shifts at the fish‐day scale. Instead, experiencing warming relative to baseline likely arose through persistence of inner habitat use, changes in microhabitat occupancy within habitats, or thermal changes within occupied habitats that did not require consistent depth adjustment. This is informative because many coastal systems can provide vertical thermal refugia through stratification. However, the availability and energetic value of deeper or cooler water depend on local bathymetry, mixing and prey distributions, and prey can remain concentrated in surface or nearshore zones (Miller & Sadro, 2003). In addition, diel and short‐duration depth movements can be muted in daily summaries. Overall, the contrast between strong temperature anomalies and weak depth anomalies suggests that event‐scale changes in thermal exposure were driven more by where fish were within the habitat mosaic (e.g., inner vs. outer areas) than by consistent shifts in depth use alone.

After controlling for strong seasonal structure in habitat use, MHW conditions were associated with increased inner‐habitat occupancy primarily for small and mean‐sized fish, whereas the largest individuals showed little change or a slight reduction. This size dependence provides a useful way to reconcile apparently contrasting findings in the literature. Warm years and low sea‐ice conditions have been associated with increased exploitation of pelagic resources and higher body condition (Anderson et al., 2023; Falardeau et al., 2022), suggesting that warm conditions can increase the profitability of outer or pelagic habitats. Conversely, Dubos et al. (2023) documented increased reliance on nearshore habitats during warmer conditions in Ungava Bay, where local knowledge emphasized the value of complex nearshore environments for predator avoidance and thermal refuge, particularly for smaller fish. Our results suggest these patterns may not be mutually exclusive: warm conditions may increase the profitability of pelagic habitats for some individuals (potentially larger fish), while simultaneously increasing nearshore reliance for smaller fish that face different constraints and risk landscapes (Thorpe, 1994).

Several mechanisms could underlie this ontogenetic divergence. Smaller fish may experience higher predation risk and therefore prioritize structurally complex inner habitats when conditions change rapidly, consistent with interpretations in Dubos et al. (2023). Smaller individuals may also benefit more from warmer temperatures if growth optima are higher at smaller body sizes (Jonsson & Jonsson, 2011; Morita et al., 2010), making inner habitats particularly profitable during MHW conditions. In contrast, large fish may already be highly inner‐biased under many conditions (limiting scope for further increases in temperature), or they may exploit outer habitats to access pelagic prey resources that become more available during warm periods (Anderson et al., 2023; Falardeau et al., 2022). Larger fish may also seek cooler waters if their optimal temperatures are lower (Mulder et al., 2020). Together, these results point toward size‐specific strategies that balance thermal opportunity, foraging profitability and risk.

Migration timing analyses indicate that interannual heatwave timing may influence marine residency in a size‐dependent way. The ecological consequences of a heatwave are likely to depend on when it occurs within the seasonal cycle, because background temperatures, prey phenology and metabolic demand differ early versus late in the marine season (Laurel et al., 2017). Entry into the marine environment was broadly similar between years, whereas marine departure occurred earlier in 2023 than 2024, and this interannual difference was driven primarily by larger individuals. This size‐specific shift is consistent with longstanding observations that larger char tend to return to freshwater earlier, which can produce a progressive decline in mean size later in the fishing season (e.g., mean lengths in the commercial catch decrease from mid‐June/July into August across stock complexes; Dempson et al., 2004). Sex‐specific migration dynamics may further contribute to these patterns. In the Nain stock complex, females are more prevalent in inshore catches than offshore, and the proportion of females in inshore catches declines over the season, consistent with earlier freshwater return by maturing females and relatively greater representation of males in more distant/offshore areas (Dempson, 1995). Against this ecological backdrop, the observed size dependence in 2023 is consistent with two non‐exclusive interpretations. First, early‐season heatwaves may have increased thermal opportunity and foraging efficiency, allowing large fish to meet energetic requirements earlier and return to freshwater sooner. This can shorten the feeding window typical of anadromous Arctic char (Dutil, 1986; Harris et al., 2020) by enhancing growth opportunities when prey availability is favourable and temperatures remain within tolerable ranges (Anderson et al., 2023; Falardeau et al., 2022). Second, early‐season heatwaves may have increased metabolic costs or imposed thermal constraints, prompting earlier departure by larger individuals if they have lower thermal optima or tighter performance margins (Gilbert et al., 2020; Gilbert & Tierney, 2018; Morita et al., 2010; Mulder et al., 2020). Evaluating these alternatives will require a tighter linkage among thermal exposure, prey fields and growth outcomes (and ideally individual sex/maturation state); however, the strong size dependence suggests that climate anomalies can alter not only when fish leave the ocean, but also which segments of the population drive year‐to‐year shifts.

Even though larger char are known to return to freshwater earlier than smaller conspecifics (Dempson et al., 2004, J. Angnatok, personal communication), Inuit local knowledge from 2023 reported larger‐than‐average Arctic char and an earlier return of large individuals that year, providing an important, independent line of evidence that aligns with the telemetry‐based pattern of size‐dependent earlier marine departure in 2023 (J. Angnatok, personal observation.). Such observations are especially valuable in highly seasonal systems where changes in availability, timing and size structure can be detected quickly through harvesting. The agreement between Inuit observations and telemetry strengthens inference that the early‐season heatwave conditions in 2023 coincided with changes in the size structure and timing of the marine feeding phase. It also highlights that heatwave impacts are not only expressed as shifts in mean behaviour, but may emerge through changes concentrated in particular size classes, patterns that are directly relevant for fisheries. Integrating Inuit knowledge with telemetry and environmental time series can therefore improve both interpretation and relevance by linking mechanistic movement data to community‐observed outcomes (e.g., changes in fish size distributions and return timing) during anomalously warm years.

Collectively, our findings suggest that the ecological consequences of MHWs for anadromous Arctic char depend on both event timing and ontogeny. Although overall heatwave exposure across the marine season was broadly similar between years, early‐season heatwaves in 2023 aligned with stronger size structuring in marine departure timing, while late‐season exposure in 2024 occurred closer to the end of the feeding period when behavioural and migratory decisions may be constrained. These patterns emphasize the importance of evaluating heatwaves in a seasonal framework rather than relying solely on annual summaries of exposure (Hobday et al., 2016; Oliver et al., 2021).

From a management perspective, size‐dependent responses imply that warm events may alter habitat use, availability and the size composition of fish encountered by fisheries in ways that vary among years depending on heatwave timing. If smaller fish increase reliance on inner habitats during MHWs, nearshore habitat quality and connectivity may become increasingly important for resilience. If large fish terminate marine occupancy even earlier during early‐season heatwave years than they usually do, fisheries may observe changes in timing and size structure consistent with Inuit observations from 2023. Because Arctic char support culturally and nutritionally important fisheries across the North, combining telemetry with Inuit knowledge and local monitoring will be essential for anticipating how continued warming and extreme events affect access, timing and population dynamics.

Several limitations highlight priorities for future work. MHW classification based on SST may not fully capture thermal conditions within different microhabitats of the study area, where temperatures can diverge from SST extracted from a fixed location. The positive relationship between regional SST and fish‐recorded internal temperature provides support for using the fixed‐location SST time series as a broad index of thermal conditions experienced by Arctic char during the marine feeding season. However, the relationship was moderate rather than exact, which is expected because internal body temperature reflects both the thermal environment encountered by fish and their behavioural habitat selection. Thus, SST is useful for identifying regional MHW conditions, but it should not be interpreted as a direct measure of the temperatures available within each habitat. This distinction is especially important in a fjord‐estuary system where local temperatures can vary with freshwater input, stratification, bathymetry and tidal mixing (Inall & Gillibrand, 2010). Although we considered using fish‐recorded temperatures to summarize habitat‐ and depth‐specific thermal conditions, detections were too sparse and unevenly distributed across habitat‐depth combinations to support robust habitat‐specific inference. Future study would be strengthened by deploying fixed temperature loggers across inner and outer habitats to directly quantify habitat‐specific thermal structure during MHW events. Nevertheless, the observed SST‐fish temperature coupling indicates that regional warming events were reflected in the temperatures experienced by tagged Arctic char during their marine feeding period.

In addition, daily fish‐day summaries may mask short‐term or diel depth responses, and receiver coverage may influence detection of fine‐scale habitat transitions. Finally, linking behaviour to energetic outcomes remains a key next step. Growth‐potential and bioenergetics models are often parameterized using juveniles or smaller‐bodied fish (Larsson et al., 2005), and may be less reliable for large, anadromous adults in the marine phase; additional validation for larger size classes would strengthen inference. Combining internal temperature time series with prey indicators, predator occurrence and condition metrics would also help evaluate indirect pathways by which heatwaves may affect char (e.g., via shifts in prey fields or predator distributions), and whether MHW‐associated warming and habitat shifts translate into enhanced growth opportunities or increased energetic stress. Finally, determining whether the size‐dependent habitat and departure shifts we observed reflect direct thermal constraints/opportunity or a prey‐mediated reorganization of foraging landscapes during MHWs by examining stomach contents and growth rates using a large‐scale mark‐recapture study would provide more insight on whether fish select habitats for thermal preferences or for prey and growth opportunity.

Arctic char in this Subarctic coastal system did not show evidence of consistent depth‐mediated avoidance during the observed MHW conditions. MHWs increased internal temperatures relative to seasonal baselines during event windows, yet did not produce consistent depth departures to cooler water. Shifts in habitat use and size‐specific migration timing were observed across years and may have reflected indirect ecological pathways, such as changes in prey availability, in addition to direct thermal exposure. These trait‐ and timing‐mediated responses indicate that MHW effects are context‐dependent but have the potential to reshape the balance of thermal opportunity and habitat trade‐offs, particularly under continued Arctic warming.

## AUTHOR CONTRIBUTIONS

J.E.D. conceived and designed the study, coordinated fieldwork, conducted all statistical analyses, designed figures and led the writing of the manuscript. D.C. secured funding, initiated the acoustic telemetry study in 2018 and contributed foundational infrastructure for the long‐term telemetry dataset. D.C. also provided logistical support during fieldwork, contributed to the conceptualization of the manuscript and provided valuable input on data interpretation and manuscript drafts. J.A. provided local knowledge and logistical support, contributed to the interpretation of ecological patterns based on Inuit Knowledge and reviewed the manuscript for relevance to the local context. T.E.V.L. and J.B.D. contributed to early discussions and provided critical feedback on data interpretation and manuscript drafts.

## FUNDING INFORMATION

Funding for this research was provided in part by ArcticNet, Crown‐Indigenous Relations and Northern Affairs Canada (CIRNAC), Polar Knowledge Canada and DFO Marine Protected and Conservation.

## Supporting information


**Figure S1.** Internal temperature spiked to ~35°C with an abnormal depth profile, indicating possible depredation, likely from a seal.
**Figure S2.** Relationship between regional sea surface temperature (SST) and fish‐recorded internal temperature for Arctic char detected in marine habitats during June–August 2023 and 2024. Fish‐day points represent one detection‐weighted internal temperature record per fish per calendar date. Daily means represent the average internal temperature across all fish detected in the marine habitat on a given date. The fitted line is shown for daily means. At the fish‐day scale, internal temperature was positively correlated with SST (*r* = 0.50, *p* ≤ 0.0001, *n* = 749 fish‐days from 26 fish). At the daily mean scale, the relationship remained positive (*r* = 0.60, *p* ≤ 0.0001, *n* = 163 calendar dates).
**Figure S3.** Fish‐recorded internal temperatures in inner and outer marine habitats during June–August 2023 and 2024. Points show detection‐weighted fish‐day‐habitat records, where Inner includes estuary and fjord detections and Outer includes coastal/headland detections. Boxplots show median and interquartile range. Values represent temperatures experienced by tagged Arctic char (*Salvelinus alpinus*) in used habitats, not fixed environmental water‐temperature measurements. The dashed line marks 14°C as a reference value for Arctic char growth‐performance context (Beuvard et al., 2022; Larsson et al., 2005).

## Data Availability

The telemetry and associated biological data supporting this study are co‐owned by the Nunatsiavut Government and are subject to Indigenous data governance and research approval processes. The authors cannot unilaterally deposit the raw data in a public repository. Requests for data access may be directed to the corresponding author and will be considered on a case‐by‐case basis in consultation with, and subject to approval by, the Nunatsiavut Government.
