# Supplementary material for: Marine heatwaves shape size‐dependent thermal exposure, habitat use and marine residency in Arctic char (Salvelinus alpinus)
Source: J Fish Biol. 2026 Jun 23;109(3):1628–44. doi: 10.1111/jfb.70543 (PMC13602086; doi:10.1111/jfb.70543)
Supplement: Supplementary file 1 [file JFB-109-1628-s001.docx]

**Supplementary Information**

Figure S1. Internal temperature spiked to ~35°C with abnormal depth profile, indicating possible depredation, likely from a seal.

Figure S2. Relationship between regional sea surface temperature (SST) and fish-recorded internal temperature for Arctic char detected in marine habitats during June-August 2023 and 2024. Fish-day points represent one detection-weighted internal temperature record per fish per calendar date. Daily means represent the average internal temperature across all fish detected in marine habitat on a given date. The fitted line is shown for daily means. At the fish-day scale, internal temperature was positively correlated with SST (r = 0.50, p ≤ 0.0001, n = 749 fish-days from 26 fish). At the daily mean scale, the relationship remained positive (r = 0.60, p ≤ 0.0001, n = 163 calendar dates).

Figure S3. Fish-recorded internal temperatures in inner and outer marine habitats during June-August 2023 and 2024. Points show detection-weighted fish-day-habitat records, where Inner includes estuary and fjord detections and Outer includes coastal/headland detections. Boxplots show median and interquartile range. Values represent temperatures experienced by tagged Arctic char (Salvelinus alpinus) in used habitats, not fixed environmental water-temperature measurements. The dashed line marks 14°C as a reference value for Arctic char growth-performance context (Larsson et al. 2005; Beuvard et al. 2022).

**Supplementary Methods**

## **Daily fish summaries and behavioural variables**

Acoustic detections were first processed using actel to remove false detections and retain biologically plausible movement records. Because individual Arctic char were sometimes detected at multiple receivers, and occasionally in more than one habitat class, within the same calendar day, detections were summarized to a daily fish-level dataset before estimating seasonal baselines and behavioural anomalies.

For each fish $i$ on date $t$, internal temperature and depth were summarized as detection-weighted daily means of receiver-level daily medians:

$$T_{it}=\frac{\sum_{j} n_{ijt}T_{ijt}}{\sum_{j} n_{ijt}}$$

$$D_{it}=\frac{\sum_{j} n_{ijt}D_{ijt}}{\sum_{j} n_{ijt}}$$

where $T_{ijt}$ and $D_{ijt}$ are the median internal temperature and depth recorded for fish $i$ at receiver or habitat unit $j$ on day $t$, and $n_{ijt}$ is the number of detections contributing to that receiver-day summary.

The total number of detections per fish-day was retained as:

$$n_{det,it}=\sum_{j} n_{ijt}$$

For marine habitat-use analyses, detections were also summarized as the number of detections occurring in inner and outer marine habitats:

$$N_{it}=n_{inner,it}+n_{outer,it}$$

Freshwater detections were excluded from inner-outer habitat-use models so that these analyses represented marine habitat use only. Because detection counts are affected by receiver placement, receiver range, habitat-specific coverage, and fish movement, these summaries should be interpreted as detection-weighted habitat use, not exact time spent in each habitat.

**Descriptive SST and fish-temperature comparison**

We assessed whether the regional sea surface temperature (SST) time series used to identify marine heatwaves reflected fish thermal experience during the marine feeding season. This analysis was restricted to marine detections during June–August 2023 and 2024; freshwater detections were excluded. Marine detections included estuary, fjord, and coastal records.

Because fish could be detected at more than one marine receiver on the same date, records were collapsed to one detection-weighted fish-day per individual per date. Daily SST values were then joined to fish-day internal temperature summaries by calendar date.

We evaluated the SST–fish temperature relationship using Pearson correlations at two scales: fish-day records and daily means averaged across all fish detected in marine habitat on each date. We also fit a descriptive mixed-effects model:

$$T_{it}=\beta_{0}+\beta_{1}SST_{t}+u_{i}+\varepsilon_{it}$$

where $T_{it}$ is the internal temperature of fish $i$ on day $t$, $SST_{t}$ is satellite-derived regional sea surface temperature on date $t$, $u_{i}$ is a fish-level random intercept, and $\varepsilon_{it}$ is residual error.

This analysis was used only to assess whether regional SST captured broad variation in fish thermal experience. It was not treated as a calibration between satellite SST and habitat-specific water temperature because implanted tags measured internal body temperature and detections reflected behaviourally selected locations.

## **Marine heatwave detection and event alignment**

Marine heatwaves were detected from a daily regional sea surface temperature time series extracted from the Copernicus Marine Environment Monitoring Service product METOFFICE-GLO-SST-L4-NRT-OBS-SST-V2. Marine heatwaves were defined following the heatwaveR implementation of Hobday et al. as periods of at least five consecutive days during which SST exceeded the seasonally varying 90th percentile threshold.

A daily climatology and threshold were generated using 2007-2022 as the baseline period. MHW events were then detected for 2023 and 2024. Each fish-day was assigned a daily MHW indicator based on whether the corresponding calendar date was classified as a MHW day.

For phase-based analyses, fish-days were aligned relative to the onset of each MHW event. The first date of each discrete MHW event was defined as:

$$t_{\mathrm{rel}}=0$$

For each fish-day, relative day was calculated as:

$$t_{\mathrm{rel}}=date_{t}-start_{k}$$

where $start_{k}$ is the onset date of MHW event $k$.

Fish-days within a 43-day window around each event onset were retained:

$$-21\leq t_{\mathrm{rel}}\leq21$$

Relative days were assigned to one of three phases: Pre: $t_{\mathrm{rel}}<0,$During: $0\leq t_{\mathrm{rel}}\leq7,$Post: $t_{\mathrm{rel}}>7$

When event windows overlapped, each calendar date was assigned to the nearest MHW onset using the smallest absolute value of $t_{\mathrm{rel}}$. If a date was exactly equidistant between two event onsets, it was assigned to the earlier event. This ensured that each fish-day received no more than one phase label.

## **Seasonal baseline estimation and anomaly calculation**

To quantify departures from expected seasonal behaviour, seasonal baselines were estimated separately for internal temperature and depth. Behavioural anomalies were then calculated as observed minus expected seasonal values:

$$T_{anom,it}=T_{it}-\hat{T}_{it}$$

$$D_{anom,it}=D_{it}-\hat{D}_{it}$$

where $T_{it}$ and $D_{it}$ are the observed daily internal temperature and depth for fish $i$ on day $t$, and $\hat{T}_{it}$ and $\hat{D}_{it}$ are the corresponding predicted seasonal baselines. Positive temperature anomalies indicate that fish were warmer than expected for that individual and time of year. Positive depth anomalies indicate that fish were deeper than expected.

Baselines were fit preferentially using non-MHW days to reduce the influence of heatwave conditions on the expected seasonal pattern. A two-tier baseline approach was used: per-fish harmonic regressions were used when individuals had sufficient data coverage, and pooled hierarchical baselines were used for fish with sparse or unstable individual baselines.

## **Per-fish harmonic seasonal baselines**

For individuals with sufficient data coverage, expected seasonal temperature and depth were estimated using per-fish harmonic regressions. These models allowed each individual to have its own seasonal baseline. For fish $i$ on day $t$, the baseline model was:

$$y_{it}=\beta_{0i}+\beta_{1i}\sin\left( \frac{2\pi DOY_{t}}{365.25} \right)+\beta_{2i}\cos\left( \frac{2\pi DOY_{t}}{365.25} \right)$$

$$+\beta_{3i}\sin\left( \frac{4\pi DOY_{t}}{365.25} \right)+\beta_{4i}\cos\left( \frac{4\pi DOY_{t}}{365.25} \right)+\delta_{\mathrm{year}\left( t \right)}+\varepsilon_{it}$$

where $y_{it}$ represents either internal temperature or depth, $DOY_{t}$ is day of year, $\delta_{\mathrm{year}\left( t \right)}$ is a fixed year effect included when observations for a fish spanned more than one year, and $\varepsilon_{it}$ is residual error.

Per-fish baselines were fit using ordinary least squares. Models were fit to non-MHW days when sufficient non-MHW observations were available. Otherwise, all available days for that fish were used to avoid fitting a seasonal curve to an extremely small subset.

A per-fish baseline was used only when an individual met all of the following criteria:

$$n_{\mathrm{days}}\geq30$$

$$n_{\mathrm{nonMHW}}\geq20$$

$$DOY_{\mathrm{span}}\geq60$$

where $DOY_{\mathrm{span}}$ is measured in days.

## **Baseline smoothness quality control**

To prevent unstable seasonal baselines for sparsely sampled individuals, per-fish baseline predictions were screened for unrealistic day-to-day changes. For consecutive observations, the maximum absolute day-to-day change in predicted baseline was calculated as:

$$\max\left| \hat{y}_{i,t}-\hat{y}_{i,t-1} \right|$$

For temperature baselines, per-fish predictions were required to satisfy:

$$\max\left| \hat{T}_{i,t}-\hat{T}_{i,t-1} \right|\leq{2.0}^{\circ}C$$

For depth baselines, predictions were required to satisfy:

$$\max\left| \hat{D}_{i,t}-\hat{D}_{i,t-1} \right|\leq5.0 m$$

Fish that failed either coverage or smoothness criteria were retained in the dataset but were assigned pooled hierarchical baselines.

## **Pooled hierarchical baseline models**

For fish that did not meet per-fish baseline criteria, or whose individual baselines failed smoothness checks, expected seasonal temperature and depth were estimated using pooled hierarchical generalized additive models. The pooled baseline model was:

$$y_{it}=\alpha+s\left( DOY_{t} \right)+b_{i}+c_{\mathrm{year}\left( t \right)}+\varepsilon_{it}$$

where $y_{it}$ is either internal temperature or depth, $s\left( DOY_{t} \right)$ is a cyclic smooth function of day of year, $b_{i}$ is a fish-level random intercept, $c_{\mathrm{year}\left( t \right)}$ is a year-level random intercept, and $\varepsilon_{it}$ is residual error.

Models were fit separately for temperature and depth using generalized additive models in mgcv with restricted maximum likelihood. Fish-days were weighted by the number of detections contributing to each daily summary:

$$w_{it}=n_{det,it}$$

Hierarchical models were fit using non-MHW fish-days when the pooled dataset contained at least 50 non-MHW fish-days. Otherwise, all fish-days were used. Final baseline predictions were generated for all fish-days, and the assigned baseline method was retained for each individual.

## **Paired within-fish phase contrasts**

Paired within-fish phase contrasts were used as a robust summary of changes in temperature and depth anomalies during and after MHW onset. For each fish, mean anomalies were calculated within each phase:

$$\overline{A}_{i,phase}$$

where $A$ represents either $T_{\mathrm{anom}}$ or $D_{\mathrm{anom}}$. For each fish, paired differences were calculated as:

$$\Delta A_{i,during-pre}=\overline{A}_{i,during}-\overline{A}_{i,pre}$$

$$\Delta A_{i,post-pre}=\overline{A}_{i,post}-\overline{A}_{i,pre}$$

Population-level effects were summarized as the median paired difference across fish:

$$\tilde{\Delta A}_{during-pre}=median\left( \Delta A_{i,during-pre} \right)$$

$$\tilde{\Delta A}_{post-pre}=median\left( \Delta A_{i,post-pre} \right)$$

Uncertainty was estimated using nonparametric bootstrap resampling of fish with replacement. For each of 2,000 bootstrap iterations, fish were resampled, median paired differences were recalculated, and 95% confidence intervals were calculated from the 2.5th and 97.5th percentiles of the bootstrap distribution. Fish contributed to a given contrast only when they had at least one fish-day in both phases being compared.

## **Daily anomaly mixed-effects models**

To test whether behavioural anomalies differed among MHW phases and whether these responses were size-dependent, linear mixed-effects models were fit separately for temperature and depth anomalies. Fork length was standardized before modelling:

$$L_{i}=\frac{\mathrm{Length}_{i}-\overline{\mathrm{Length}}}{SD\left( \mathrm{Length} \right)}$$

where $L_{i}$ is standardized fork length for fish $i$. The temperature anomaly model was:

$$T_{anom,it}=\beta_{0}+\beta_{1}I_{during,it}+\beta_{2}I_{post,it}+\beta_{3}L_{i}$$

$$+\beta_{4}I_{during,it}L_{i}+\beta_{5}I_{post,it}L_{i}+u_{i}+v_{\mathrm{year}\left( t \right)}+\varepsilon_{it}$$

The depth anomaly model used the same structure:

$$D_{anom,it}=\beta_{0}+\beta_{1}I_{during,it}+\beta_{2}I_{post,it}+\beta_{3}L_{i}$$

$$+\beta_{4}I_{during,it}L_{i}+\beta_{5}I_{post,it}L_{i}+u_{i}+v_{\mathrm{year}\left( t \right)}+\varepsilon_{it}$$

where $I_{during,it}$ and $I_{post,it}$ are indicator variables for MHW phase, with the pre-MHW phase used as the reference category. $u_{i}$ is a random intercept for fish identity, $v_{\mathrm{year}\left( t \right)}$ is a random intercept for year, and $\varepsilon_{it}$ is residual error.

Estimated marginal means were calculated at three representative body sizes:

$$L_{i}=-1, 0, +1$$

corresponding to approximately one standard deviation below the mean, the mean, and one standard deviation above the mean. Tukey-adjusted pairwise contrasts among phases were calculated at each representative body size. Phase-specific slopes of anomaly versus length were also estimated to aid interpretation of phase-by-length interactions.

## **Marine habitat-use model**

Marine habitat use was modelled using the number of detections assigned to inner and outer habitats for each fish-day. Freshwater detections were excluded from this analysis. The response was the number of inner detections out of the total number of marine detections:

$$n_{inner,it}\sim Binomial\left( N_{it},p_{it} \right)$$

$$N_{it}=n_{inner,it}+n_{outer,it}$$

where $p_{it}$ is the probability that detections for fish $i$ on day $t$ occurred in inner habitat. A binomial generalized additive mixed model with a logit link was fit as:

$$\mathrm{logit}\left( p_{it} \right)=\beta_{0}+\beta_{1}I_{MHW,it}+\beta_{2}L_{i}+\beta_{3}I_{MHW,it}L_{i}$$

$$+s\left( DOY_{t} \right)+u_{i}+v_{\mathrm{year}\left( t \right)}$$

where $I_{MHW,it}$ indicates whether date $t$ was classified as a MHW day, $L_{i}$ is standardized fork length, $s\left( DOY_{t} \right)$ is a cyclic smooth function of day of year, $u_{i}$ is a fish-level random intercept, and $v_{\mathrm{year}\left( t \right)}$ is a year-level random intercept.

The cyclic smooth of day of year was included to account for strong seasonal structure in marine habitat use. The MHW-by-length interaction tested whether the effect of MHW conditions on inner-habitat use differed among body sizes.

Marginal predictions of inner-habitat use were generated at:

$$L_{i}=-1, 0, +1$$

Predicted probabilities were averaged over the observed day-of-year distribution while excluding fish and year random effects to obtain population-level predictions. Uncertainty around marginal predicted probabilities was estimated using cluster bootstrap resampling of fish.

Size-specific MHW effects were evaluated as odds ratios comparing MHW and non-MHW days:

$$OR=exp\left( \Delta\mathrm{logit} \right)$$

where $OR>1$ indicates higher odds of inner-habitat use on MHW days, and $OR<1$ indicates lower odds of inner-habitat use on MHW days. Because the response is based on detection counts, this model evaluates detection-weighted inner versus outer habitat use rather than exact residence time.

## **Migration timing model**

Marine entry and return timing were indexed using detections in non-freshwater habitats. For each fish-year, marine entry was defined as the first non-freshwater detection date, and return timing was indexed as the last non-freshwater detection date. The span between first and last non-freshwater detections was used as a proxy for marine feeding duration.

Fish-years were retained for migration timing analysis when they had a first non-freshwater detection on or before 30 June, at least one non-freshwater detection, and a non-freshwater detection span greater than 20 days.

Return timing was modelled as the day of year of the last non-freshwater detection:

$$Y_{iy}=\beta_{0}+\beta_{1}I_{2024,y}+\beta_{2}L_{i}+\beta_{3}I_{2024,y}L_{i}+\varepsilon_{iy}$$

where $Y_{iy}$ is the last non-freshwater detection day of year for fish $i$ in year $y$, $I_{2024,y}$ is an indicator for 2024, $L_{i}$ is standardized fork length, and $\varepsilon_{iy}$ is residual error.

A fish-level random intercept was initially evaluated because some individuals contributed observations in more than one year. However, this model produced a singular fit, indicating that the fish-level random-effect variance was effectively zero. Therefore, the corresponding linear model was retained.

Model support for additive versus year-by-length interaction forms was compared using AIC. Interaction-aware estimated marginal means were calculated at:

$$L_{i}=-1, 0, +1$$

to compare return timing between years for small, mean-sized, and large fish.

## **Model interpretation and reporting**

For all models involving standardized length, effects were interpreted at small, mean-sized, and large body sizes corresponding to:

$$L_{i}=-1, 0, +1$$

For phase-based anomaly models, contrasts among pre, during, and post phases were interpreted as changes in behaviour relative to expected seasonal baselines rather than changes in raw temperature or raw depth.

For habitat-use models, results were reported as predicted probabilities of inner-habitat use and as odds ratios comparing MHW and non-MHW days. Because habitat-use responses were based on acoustic detection counts, language was restricted to “detection-weighted habitat use”, “probability of inner-habitat detections”, or “relative use of inner versus outer habitats”, rather than “proportion of time spent” unless supported by additional residence-time analyses.

For migration timing models, last non-freshwater detection was interpreted as a proxy for marine departure or upstream return timing, rather than a direct observation of river entry, because receiver coverage and detection probability can affect the exact timing of last marine detection.
